# Editorial: Improving aneurysmal Subarachnoid hemorrhage management, what's new?

**DOI:** 10.3389/fneur.2024.1419224

**Published:** 2024-05-29

**Authors:** Valentina Tardivo, Emanuela Crobeddu, Massimo Del Sette, Luca Valvassori, Marcello Egidi

**Affiliations:** ^1^Department of Neurosurgery, San Carlo Borromeo Hospital, Milan, Italy; ^2^Department of Neurosurgery, Azienda Ospedaliero Universitaria Maggiore della Carità, Novara, Italy; ^3^Unit of Neurology, San Martino Hospital (IRCCS), Genoa, Italy; ^4^Department of Neuroradiology, ASST Santi Paolo e Carlo, Milan, Italy

**Keywords:** aneurysmal subarachnoid hemorrhage (aSAH), vasospasm, Hydrocephalus - complications, delayed cerebral ischemia (DCI), aneuryms

Despite significant progress in critical care, aneurysmal subarachnoid hemorrhage (aSAH) still represents a severe form of stroke with a high mortality rate.

Prehospital mortality rates for aSAH range from 22% to 26%. Although hospital mortality rates have shown stability (13.7% in 2006 to 13.1% in 2018 (United States) (1) and 19%−20% in 2021 (global) (2), population-based studies indicate a decline in the overall case-fatality rates (−1.5%/year between 1980 and 2020) albeit with substantial inter-country variability (3, 4).

While aSAH incidence varies between populations, nearly half of all survivors experience some level of persistent neurological deficit. This burden is particularly high because, unlike other types of stroke, aSAH affects patients in their working years, with a mean age of 55 years (1). Moreover, aSAH imposes significant economic costs, with estimated inpatient hospital charges ranging from $373 353.94 to $530 544.77 [for those individuals with aSAH who develop delayed cerebral ischemia (DCI)] in the United States (5).

Managing aSAH is a multidisciplinary challenge that extends beyond treating and securing the bleeding aneurysm, as outcomes are influenced by complications like vasospasm, hydrocephalus, and delayed ischemic deficit.

The main purpose of this Research Topic was to gather publications that focus on the advancement and update of management criteria and therapeutic strategies to improve the daily multidisciplinary care of aSAH.

When this Research Topic was issued, the most recent available guidelines dated back to 2012 and covered literature up to May 2010 (A revision, from a European standpoint, was provided by the Guidelines Committee of the European Stroke Organization in 2013) (6, 7).

The new aSAH management guidelines published by the American Heart Association/American Stroke Association in June 2023 (8) have updated many recommendations based on new evidence, although some controversy persists.

For instance, it is globally accepted that nimodipine is a mainstay of treatment after aneurysmal subarachnoid hemorrhage, as it has been shown to reduce the risk of delayed cerebral ischemia with consequent improved functional outcomes after aSAH. Therefore, guidelines advocate early initiation of enteral nimodipine. However recent studies suggest individual variability in its absorption in aSAH patients, potentially affecting the prognosis (Mahmoud et al.).

Despite advances, in-hospital mortality and severe disability rates in aSAH patients remain of concern, with a subset of patients at higher risk of complications and poor outcomes (Liu et al.; Liu and Wang; Bögli et al.; Scibilia et al.).

There is growing interest in recognizing sex-specific extra-cerebral complications, which could lead to the development of more tailored monitoring and therapeutic strategies to reduce mortality and disability from aSAH.

For example, it has been reported that women appear to be at greater risk of cardiac and infectious complications than men, and, together with patients with a history of cardiac disorders, they may benefit from personalized management when at risk for symptomatic vasospasm/DCI. In these patients, either a closer evaluation for a new cardiac injury or possibly lower blood pressure targets should be therefore considered (Bögli et al.).

In a retrospective study by Hu, age, blood glucose (>7.22 mmol/L), Glasgow Coma Scale, and red blood cell distribution width-SD were proven to be independent risk factors for hospital-acquired pneumonia in aSAH patients (HAP) (Hu et al.). In this respect, it has been previously reported that HAP in aSAH is associated with worse long-term outcomes as respiratory complications compromise air exchange and aggravate hypoxia, worsening brain injury (9).

Early identification of higher-risk aSAH patients could facilitate the application of individualized management and treatment strategies to improve outcomes and reduce the social costs of this devastating disease. Integration of these considerations into the emergency care of low grade aSAH patients, along with clinical and anamnestic information, may support the multidisciplinary neurovascular team in making clinical decisions and engaging in realistic discussions with patients' caregivers.

While progress has been made, several aspects continue to challenge us. Despite a seeming decline in the overall incidence and prevalence of aSAH, persistently high in-hospital and prehospital mortality rates and an increased incidence in the elderly population require refinement of therapies and practice standards for the management of aSAH patients and, hopefully, non-invasive and low-cost tests to better identify patients at high risk for poor outcomes.

Advancements in the diagnostic process, management strategies, and understanding of the complex mechanism of injury following aSAH hold promise for improving patient prognosis in the future.

## Author contributions

VT: Writing—original draft, Writing—review & editing. EC: Writing—review & editing. MD: Methodology, Supervision, Writing—review & editing. LV: Conceptualization, Methodology, Supervision, Writing—review & editing. ME: Conceptualization, Methodology, Supervision, Writing—review & editing.
